# Supplementary material for: CEBPA restricts alveolar type 2 cell plasticity during development and injury-repair
Source: Nat Commun. 2024 May 16;15:4148. doi: 10.1038/s41467-024-48632-3 (PMC11099190; doi:10.1038/s41467-024-48632-3)
Supplement: Supplementary file 1 — Supplementary Information [file 41467_2024_48632_MOESM1_ESM.pdf]

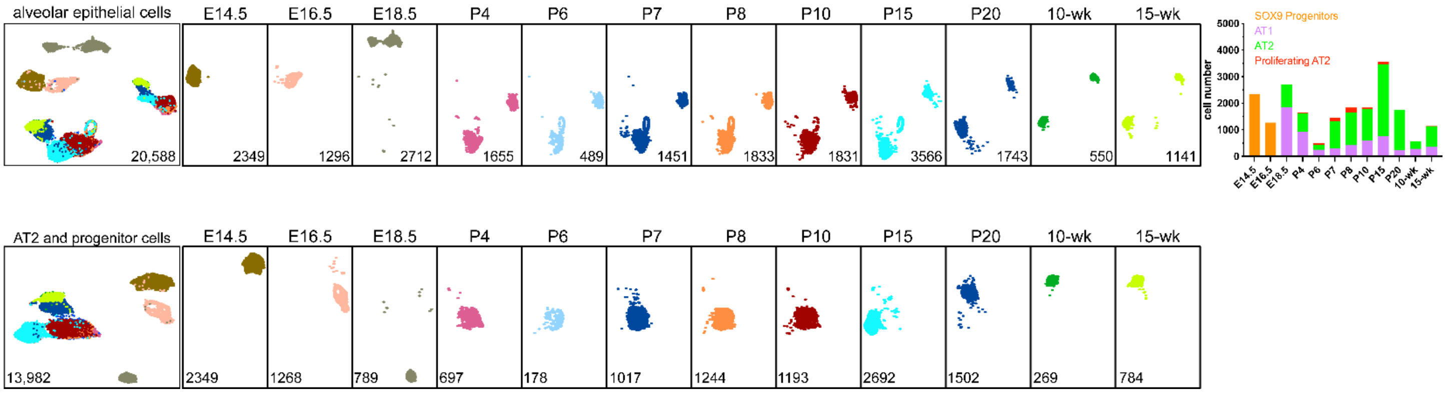

Supplementary Figure 1. UMAPs for individual time points and associated cell numbers and proportions in Fig. 1A, 1B.

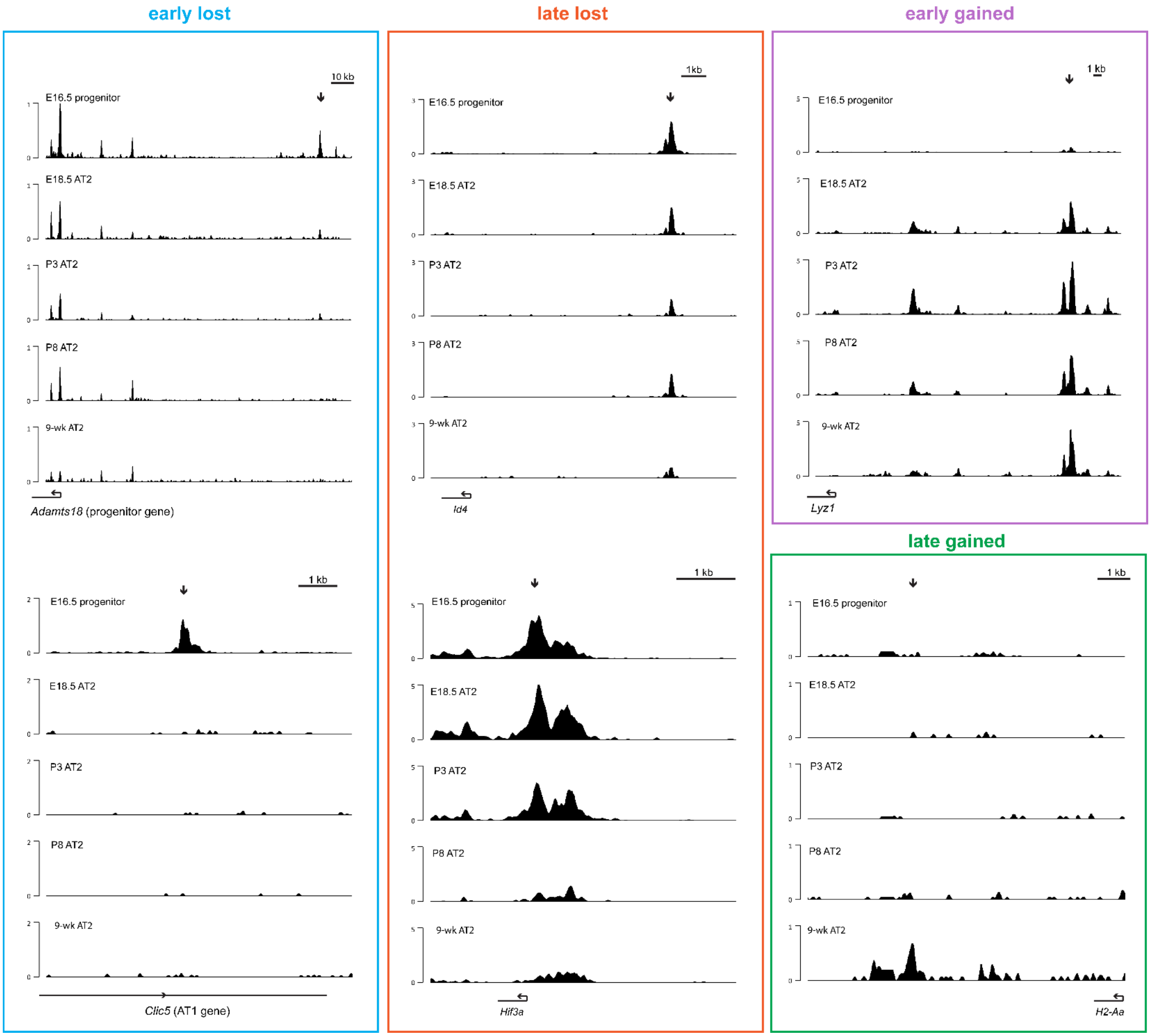

Supplementary Figure 2. Representative genomic snapshots of the 4 categories of peaks in Fig. 1D.

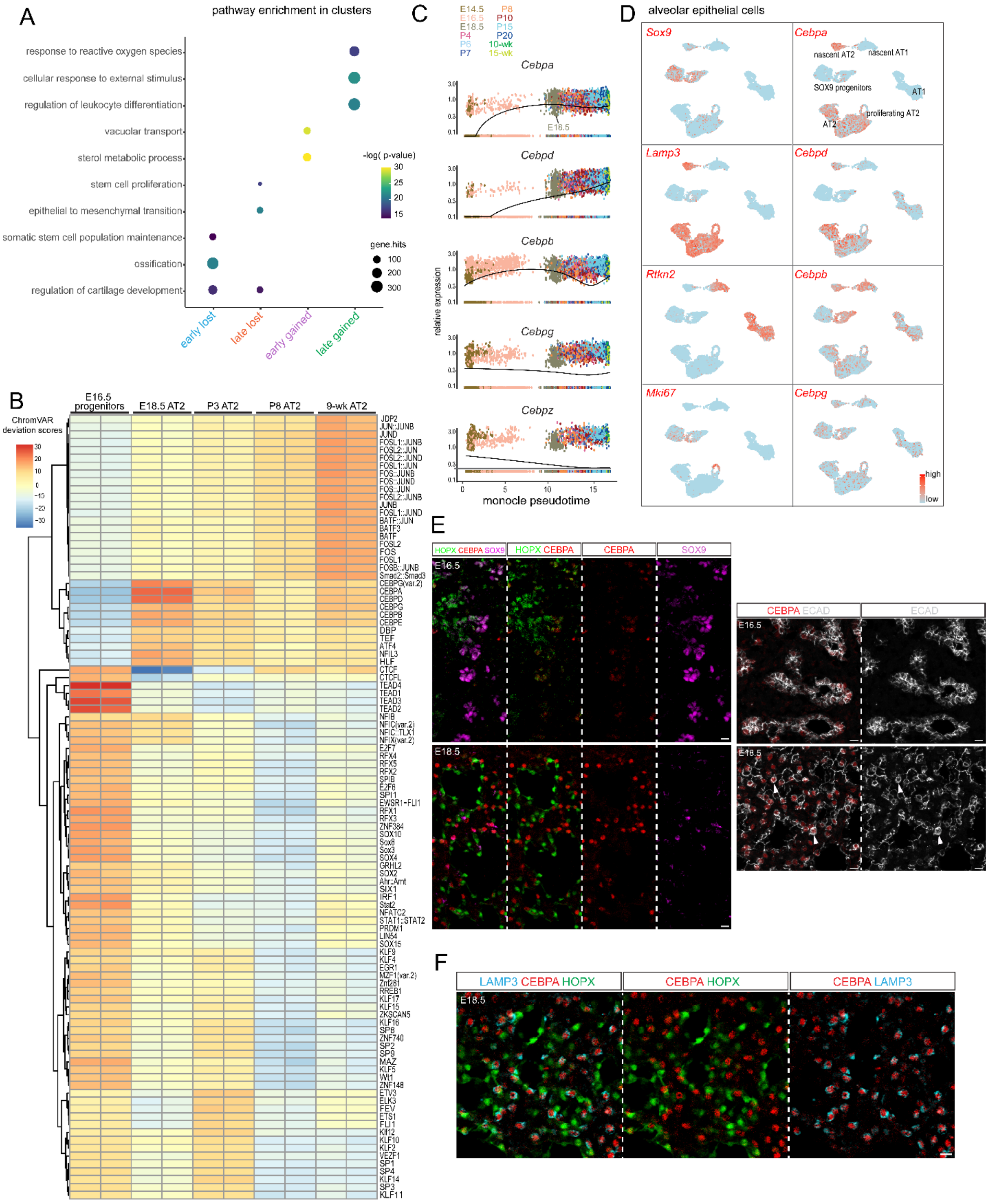

Supplementary Figure 3. Time-course analysis of AT2 cell development and CEBPA expression. (**A**) Biological process GO terms for the nearest genes of the 4 ATAC-seq clusters in Fig. 1D. (**B**) Heatmap of ChromVAR deviation scores to the 100 most variable motifs across time. (C) Monocle pseudotemporal expression changes of 5 CEBP family members across 12 time points in Fig. 1B. *Cebpa*, but not other CEBP genes, reaches maximal expression upon AT2 specification. *Cebpe* is excluded due to lack of expression in alveolar epithelial cells. (D) Feature plots of Fig. 1A showing robust expression of *Cebpa*, but not other CEBP genes, in nascent AT2 cells. (E) Left: confocal images showing CEBPA is not expressed in SOX9 progenitors nor HOPX+ AT1 cells as SOX9 progenitors differentiate into AT1 and AT2 cells from E16.5 to E18.5. Right: confocal images showing CEBPA in cuboidal cells outlined by ECAD at E18.5 (arrowhead). (F) Confocal images showing CEBPA is expressed in LAMP3+ AT2 cells but not HOPX+ AT1 cells. Scale: 10 μm . Source data are provided in Table S1.

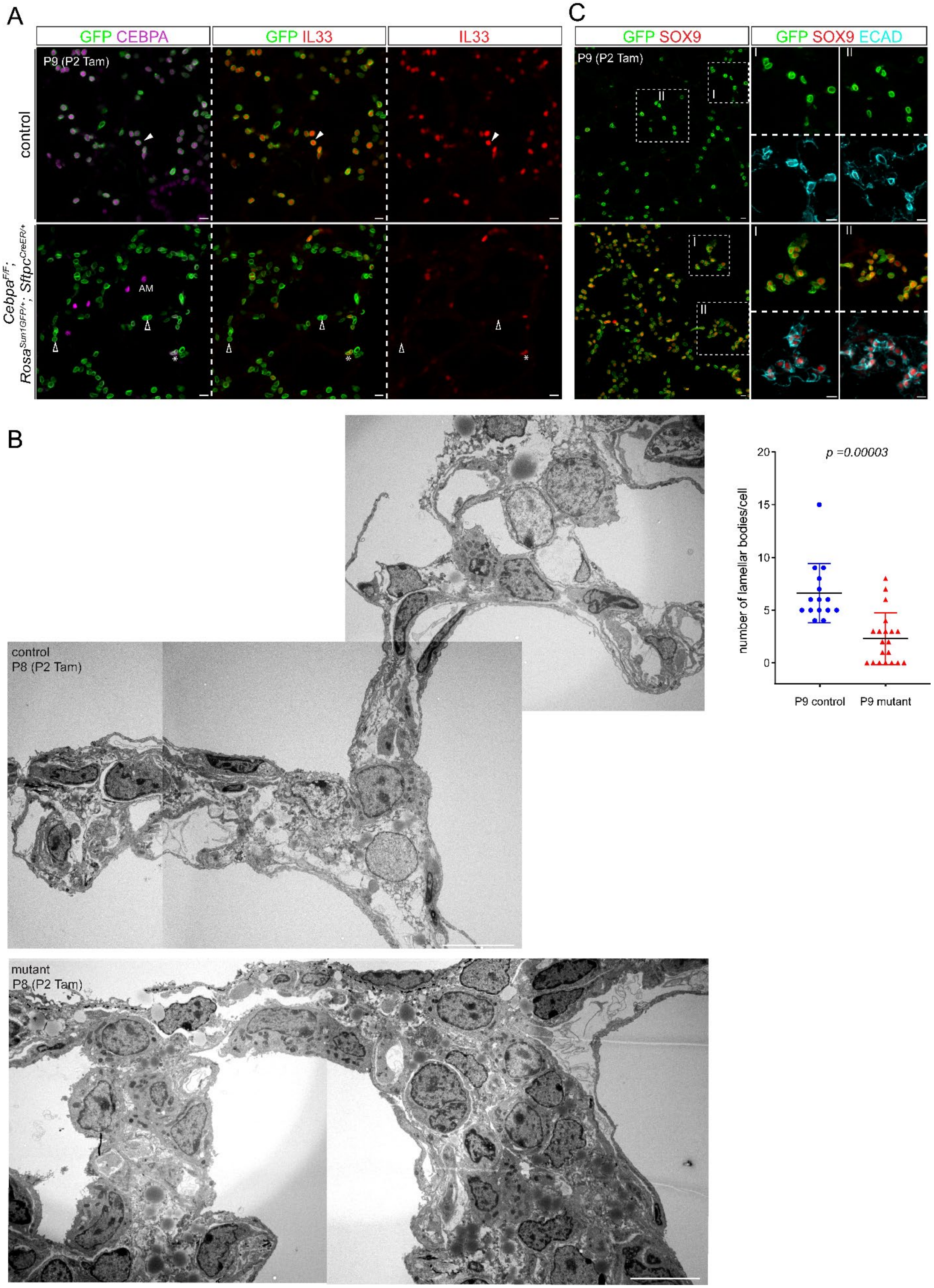

Supplementary Figure 4. Characterization of neonatal Cebpa mutant AT2 cells. (**A**) Confocal images showing loss of CEBPA and IL33 in GFP+ recombined neonatal mutant AT2 cells (filled vs open arrowhead). AM, alveolar macrophage; *, escaper of *Cebpa* deletion still expressing IL33. (**B**) Stitched TEM images showing higher cell density in the mutant. Quantification of lamellar bodies for Fig. 2C. *P* values were calculated using two-tailed Student's t-test. (C) Confocal images showing adjoining (ECAD) ectopic SOX9 cells in the mutant, resembling SOX9 progenitors at embryonic branch tips. Scale: 10 µm. Source data are provided in Table S2.

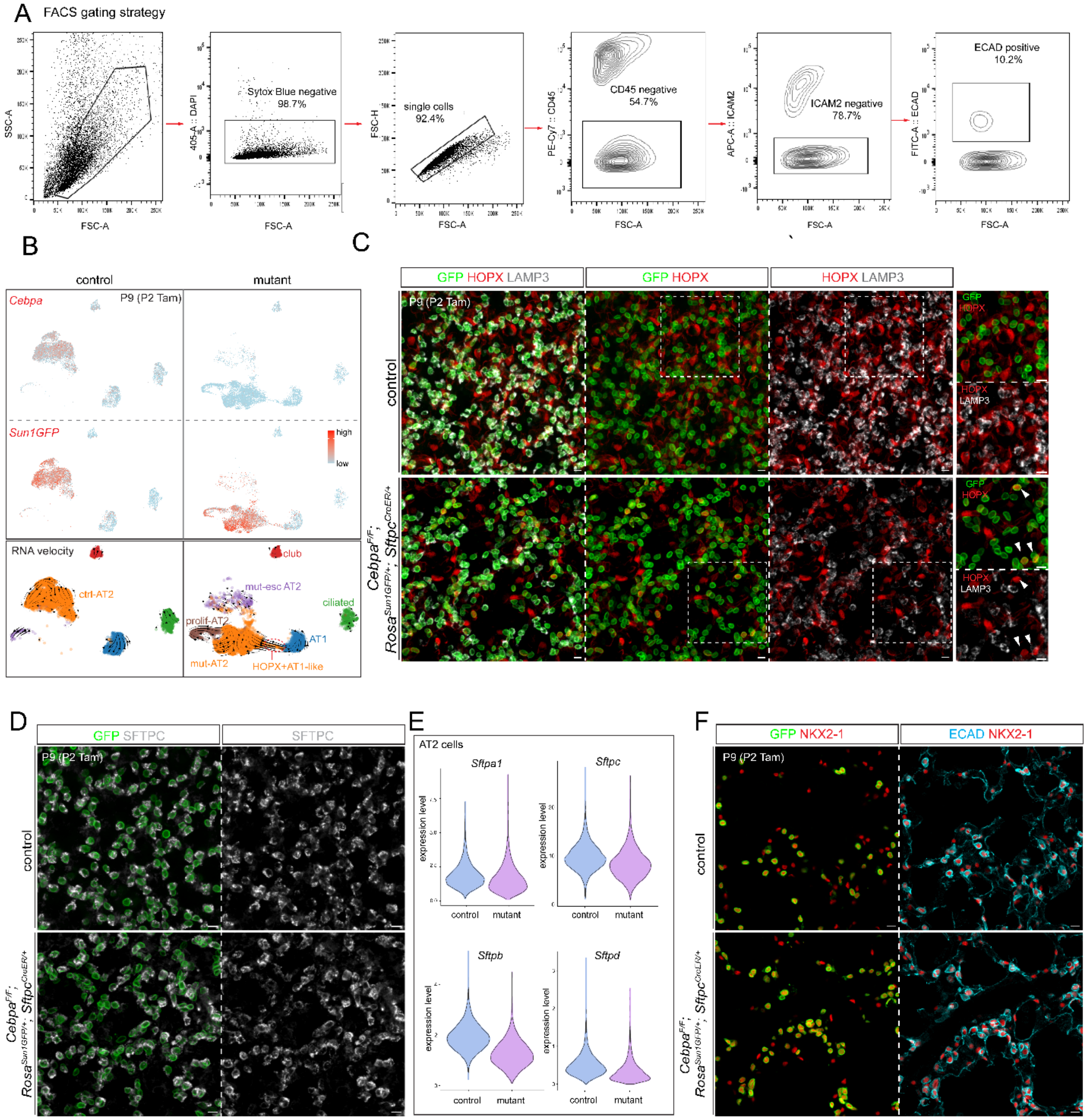

Supplementary Figure 5. Multiome and staining of neonatal Cebpa mutant AT2 cells. (**A**) FACS gating strategy to purify lung epithelial cells. (**B**) Top: split feature plots of Fig. 3C to better visualize the control and mutant. Bottom: RNA velocity analysis showing a predicted trajectory from AT2 to AT1 cells through a bridging population specifically in the mutant. (**C**) Confocal images showing that HOPX+ mutant AT2 cells do not express LAMP3 (arrowhead). (**D**) Confocal images showing persistent, albeit somewhat lower, SFTPC in mutant AT2 cells. (**E**) Violin plots of control and mutant AT2 cells in Fig. 3A showing a small decrease in surfactant gene expression. (**F**) Confocal images showing normal NKX2-1 expression in mutant AT2 cells. Scale: 10 µm.

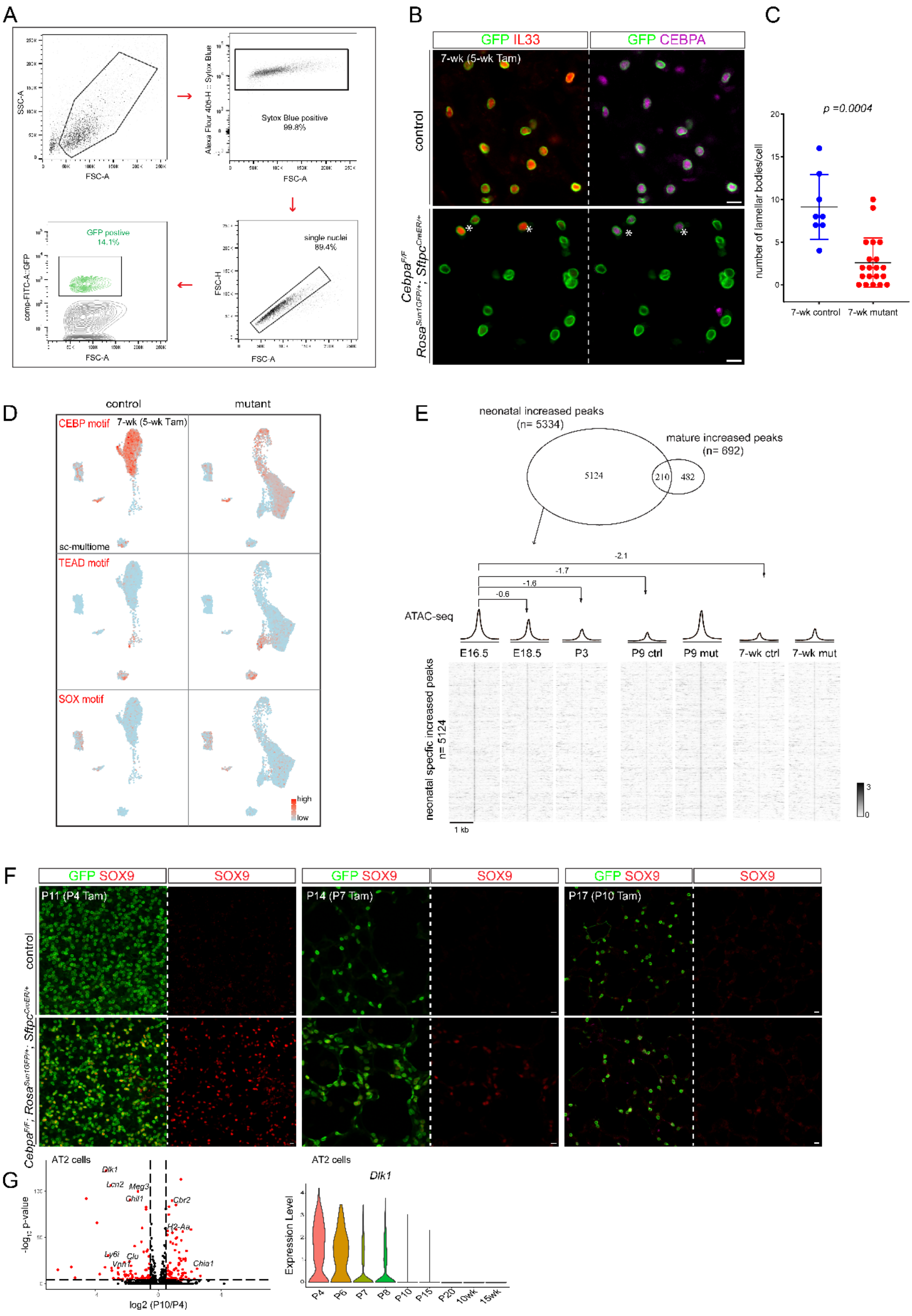

Supplementary Figure 6. ChIP-seq and comparison of neonatal vs mature Cebpa mutant AT2 cells. (**A**) FACS gating strategy to purify AT2 nuclei for ChIP-seq. (**B**) Confocal images showing loss of CEBPA and IL33 in GFP+ recombined mature mutant AT2 cells, except for escapers of deletion (asterisk). Tam, 3 mg tamoxifen. Scale: 10 μm. (**C**) Quantification of lamellar bodies in mature AT2 cells for Fig. 5B. *P* values were calculated using two-tailed Student's t-test. (**D**) Feature plots of motif activities for Fig. 5C. (**E**) Top: Venn diagram comparison of increased peaks in neonatal (Fig. 3F) vs mature (Fig. 5G) mutant AT2 cells. Bottom: heatmaps and profile plots showing that neonatal-specific increased peaks gradually lose accessibility (log2 fold change) from E16.5 to 7-wk. (**F**) Confocal images showing P4 but not P10 deletion of *Cebpa* in AT2 cells leads to SOX9 activation. P7 deletion leads to weak SOX9 activation. Tam, 250 μg for P4 and 400 μg for P7 and P10 tamoxifen. Scale: 10 μm. (**G**) Left: volcano plot (two-tailed, non-parametric Wilcoxon rank sum test) comparison of P4 and P10 scRNA-seq of AT2 cells. Right: violin plot of *Dlk1* expression as AT2 cells mature. Source data are provided in Table S5.

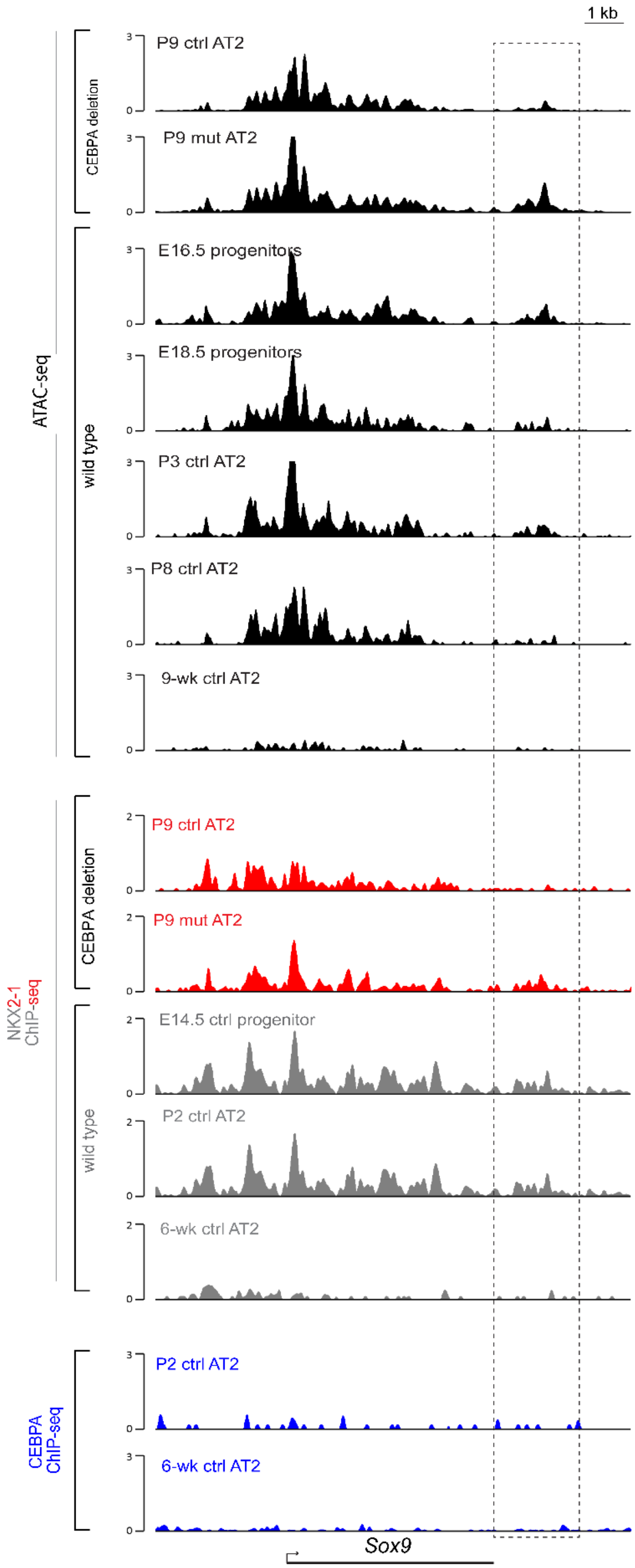

Supplementary Figure 7. Coverage plots showing a putative regulatory region 3' to Sox9 (box) that opens with more NKX2-1 binding upon Cebpa deletion, gradually closes and loses NKX2-1 binding during AT2 cell development in wild type lungs, and does not have CEBPA binding.

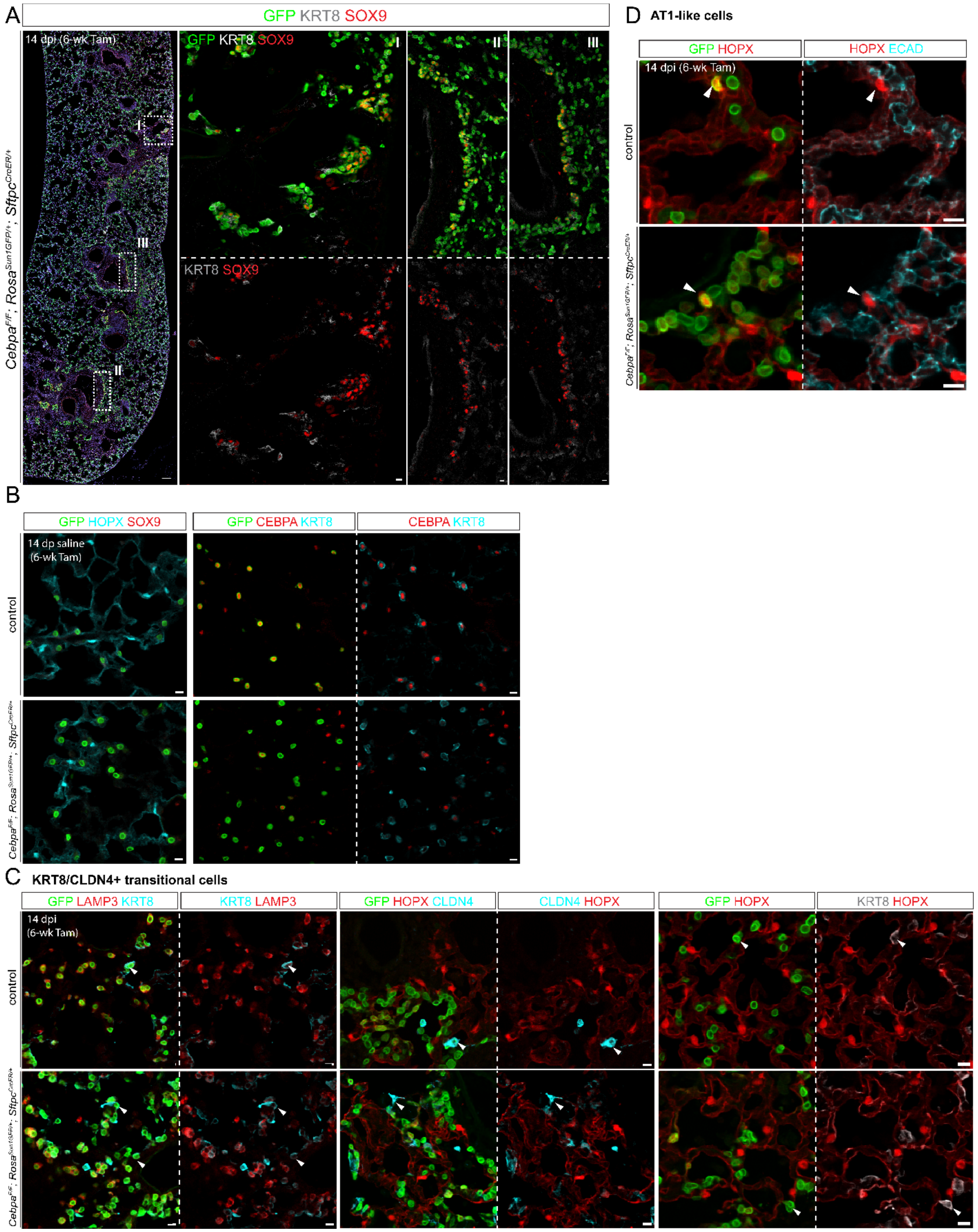

Supplementary Figure 8. Characterization of control and Cebpa mutant lungs exposed to Sendai virus or saline. (**A**) Confocal images showing SOX9 reactivation, distinct from KRT8 expression, near lobe edges (I) and airways/macro-vessels (II, III). Scale: 100 µm (10µm for insets). (**B**) Confocal images showing no SOX9 reactivation, HOPX expression, nor high KRT8 expression upon saline administration in control and mutant lungs. Baseline KRT8 expression is present in all AT2 cells. Scale: 10 µm. (**C**) Confocal images showing that KRT8/CLDN4+ cells have low LAMP3 and no HOPX (arrowhead). Scale: 10 µm. (**D**) Confocal images showing that AT1-like cells expressing HOPX (arrowhead) are no longer cuboidal (ECAD). Scale: 10 µm.

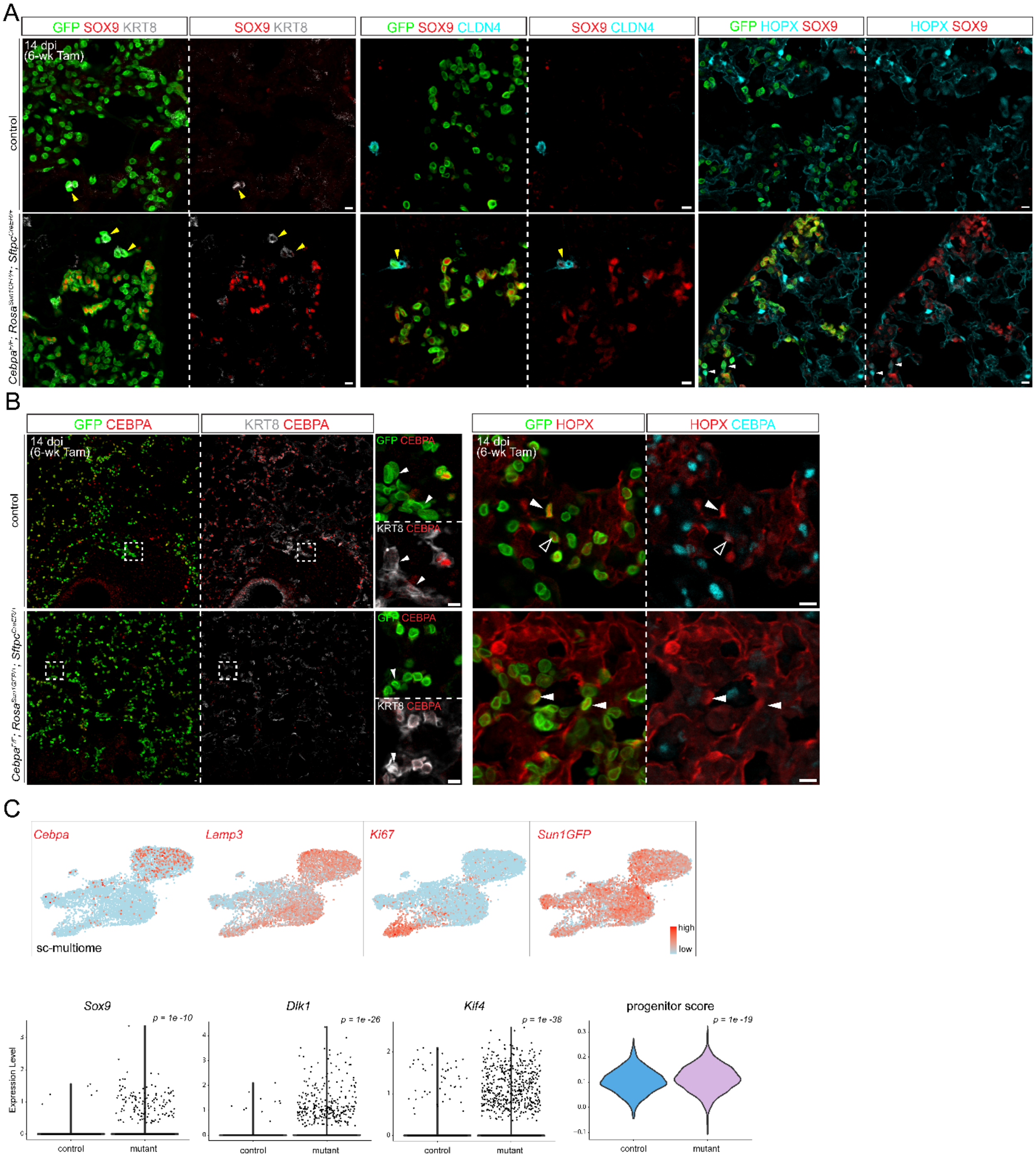

Supplementary Figure 9. Additional characterization of SOX9 and CEBPA in infected control and Cebpa mutant lungs. (**A**) Confocal images show that reactivated SOX9 in infected mutant AT2 cells is not in KRT8/CLDN4+ cells (yellow arrowhead) nor AT1-like cells (HOPX+; white arrowhead). Scale: 10 μm. (**B**) Confocal images showing loss of CEBPA in KRT8/CLDN+ cells (left) and AT1-like cells (HOPX+; arrowhead) (right) even in the control lung. Open arrowhead, low CEBPA. Scale: 10 μm. (**C**) Top: feature plots for Fig. 6G showing loss/reduction of *Cebpa* and *Lamp3* in KRT8/CLDN4+ and AT1-like cells even in the control lung. Bottom: violin plots for Fig. 6G showing activation of progenitor genes *Sox9*, *Dlk1*, and *Kif4* in *Cebpa* mutant AT2 cells. *P* value calculated using two-tailed, non-parametric Wilcoxon rank sum test. Source data are provided in Table S6.
