# Supplementary material for: CEBPA restricts alveolar type 2 cell plasticity during development and injury-repair
Source: Nat Commun. 2024 May 16;15:4148. doi: 10.1038/s41467-024-48632-3 (PMC11099190; doi:10.1038/s41467-024-48632-3)
Supplement: Supplementary file 3 — Reporting Summary [file 41467_2024_48632_MOESM3_ESM.pdf]

nature portfolio

Corresponding author(s): Jichao Chen

Last updated by author(s): Apr 25, 2024

# Reporting Summary

Nature Portfolio wishes to improve the reproducibility of the work that we publish. This form provides structure for consistency and transparency in reporting. For further information on Nature Portfolio policies, see our Editorial Policies and the Editorial Policy Checklist.

## Statistics

For all statistical analyses, confirm that the following items are present in the figure legend, table legend, main text, or Methods section.

| n/a | Confirmed | |
|---|---|---|
| [ ] | [x] | The exact sample size (*n*) for each experimental group/condition, given as a discrete number and unit of measurement |
| [ ] | [x] | A statement on whether measurements were taken from distinct samples or whether the same sample was measured repeatedly |
| [ ] | [x] | The statistical test(s) used AND whether they are one- or two-sided<br>*Only common tests should be described solely by name; describe more complex techniques in the Methods section.* |
| [x] | [ ] | A description of all covariates tested |
| [ ] | [x] | A description of any assumptions or corrections, such as tests of normality and adjustment for multiple comparisons |
| [x] | [ ] | A full description of the statistical parameters including central tendency (e.g. means) or other basic estimates (e.g. regression coefficient) AND variation (e.g. standard deviation) or associated estimates of uncertainty (e.g. confidence intervals) |
| [x] | [ ] | For null hypothesis testing, the test statistic (e.g. *F*, *t*, *r*) with confidence intervals, effect sizes, degrees of freedom and *P* value noted<br>*Give P values as exact values whenever suitable.* |
| [x] | [ ] | For Bayesian analysis, information on the choice of priors and Markov chain Monte Carlo settings |
| [x] | [ ] | For hierarchical and complex designs, identification of the appropriate level for tests and full reporting of outcomes |
| [x] | [ ] | Estimates of effect sizes (e.g. Cohen's *d*, Pearson's *r*), indicating how they were calculated |

*Our web collection on statistics for biologists contains articles on many of the points above.*

## Software and code

Policy information about availability of computer code

| | |
|---|---|
| Data collection | FACS was performed on on Aria II Cell sorter with a 70µm nozzle at 4°C. Fluorescence images were collected on using Olympus FV1000 confocal microscope and quantified with the Imaris software (version 7.7.2). |
| Data analysis | All bioinformatics analyses were performed using previously published and publicly available packages. Sequencing experiments were analyzed using R (version 4.1.1) and Python (version 3.11.5).<br>ScRNA-seq time course analysis was generated using Seurat (version 4.1), Monocle (version 2.22.0), pheatmap (version 1.0.12) and ggpubr (version 0.6.0).<br>ChIP-seq were analyzed using using: Fastqc (version 0.11.8), Trimmomatic (version 0.33), Bowtie (version 2.4.1), Picard (version 2.9.0), Samtools (version 1.15), MACS2( version 2.4.1), Diffbind (version 3.4.11), Bedtools(version 2.30.0), Bamcoverage (version 3.3.2), ChIPseeker (version 1.3), ChIPpeakAnno (version3.2) and GREAT (Version 4.0.4). Motif analysis was performed using: Homer (version 4.10), ChromVAR (version 1.16.0) and JASPAR (2020 Version).<br>Pseudobulk ATAC-seq time course analysis was generated using Signac (version 1.9), Sinto (version 0.4.0), MACS2 (version 2.1.2), Rsubread (version 2.8.2), DEseq2 (version 1.34.0), Diffbind (version 3.4.11), (GREAT, version 4.0.4)<br>scMultione were analyzed using: cellranger-arc ( version 2.0.0) and the R's (version 4.1.1) packages: Seurat(version 4.3), Signac (version 1.9), ChromVAR (version 1.16.0). RNA velocity was analyzed in Python(version 3.11.5) using velocyto((version 0.17.17) and scvelo (version 0.3.1).<br>Custom code for the analysis are provided in Supplemental software File1 that provide R script codes run for the analysis so that others can reproduce our analysis.<br>Heatmaps and genomic trackers were generated using EaSeq (version 1.2)<br>Cell sorting was analyzed using FlowJo (version10) |

For manuscripts utilizing custom algorithms or software that are central to the research but not yet described in published literature, software must be made available to editors and reviewers. We strongly encourage code deposition in a community repository (e.g. GitHub). See the Nature Portfolio guidelines for submitting code & software for further information.

## Data

Policy information about availability of data

All manuscripts must include a data availability statement. This statement should provide the following information, where applicable:

- Accession codes, unique identifiers, or web links for publicly available datasets
- A description of any restrictions on data availability
- For clinical datasets or third party data, please ensure that the statement adheres to our policy

The wild-type scRNA-seq data have been previously published 8 and are available at Gene Expression Omnibus under the accession number GEO: GSE158192 [https://www.ncbi.nlm.nih.gov/geo/query/acc.cgi?acc=GSE158192]. The wild-type scATAC-seq data used in the time course analysis are available through GEO: GSE264098 [https://www.ncbi.nlm.nih.gov/geo/query/acc.cgi?acc=GSE264098]. The P3 scATAC-seq data set was previously published and available through GEO: GSM4504962 [https://www.ncbi.nlm.nih.gov/geo/query/acc.cgi?acc=GSM4504962 ). The ChIP-seq data in this study is available at the GEO: GSE247271 [https://www.ncbi.nlm.nih.gov/geo/query/acc.cgi?acc=GSE247271]. 10-wk NKX2-1 AT1 ChIP-seq is available under GEO: GSE158205 [https://www.ncbi.nlm.nih.gov/geo/query/acc.cgi?acc=GSE158205]. The scMultiome data is available at the GEO:GSE247130, [https://www.ncbi.nlm.nih.gov/geo/query/acc.cgi?acc=GSE247130]

## Research involving human participants, their data, or biological material

Policy information about studies with human participants or human data. See also policy information about sex, gender (identity/presentation), and sexual orientation and race, ethnicity and racism.

| | |
|---|---|
| Reporting on sex and gender | NA |
| Reporting on race, ethnicity, or other socially relevant groupings | NA |
| Population characteristics | NA |
| Recruitment | NA |
| Ethics oversight | NA |

Note that full information on the approval of the study protocol must also be provided in the manuscript.

# Field-specific reporting

Please select the one below that is the best fit for your research. If you are not sure, read the appropriate sections before making your selection.

☒ Life sciences ☐ Behavioural & social sciences ☐ Ecological, evolutionary & environmental sciences

For a reference copy of the document with all sections, see nature.com/documents/nr-reporting-summary-flat.pdf

# Life sciences study design

All studies must disclose on these points even when the disclosure is negative.

| | |
|---|---|
| Sample size | For ChIP-seq and scMultiome two biological replicates is the standard in the field, while for single-cell data it is acceptable to perform it one time on thousands of cells using 10X Genomics.<br>For phenotype analysis by imaging, no power analysis for sample size was carried out, but our assays rely upon cellular readouts of hundreds to thousands of cells per mouse and 2-3 mice per experimental group, which is sufficient to ensure reproducible results based on prior experience. |
| Data exclusions | Data was only excluded if technical errors during the experiment were detected. |
| Replication | All confocal images are representative of at least 3 imaging fields of each sample and at least 3 sets of control and mutant lungs.<br>Hundreds to thousands of cells were quantified in each comparison.<br>ChIP-seq experiments was carried out twice using biological replicates.<br>ScMultiome experiments were carried out once for each condition. |
| Randomization | Animals were genotyped to identify control and mutant mice. No randomization is needed. |

| | |
|---|---|
| Blinding | Blinding was not used because the complexity in the genetics and the obvious molecular phenotypes. |

# Reporting for specific materials, systems and methods

We require information from authors about some types of materials, experimental systems and methods used in many studies. Here, indicate whether each material, system or method listed is relevant to your study. If you are not sure if a list item applies to your research, read the appropriate section before selecting a response.

## Materials & experimental systems

| n/a | Involved in the study |
|---|---|
| ☐ | ☒ Antibodies |
| ☒ | ☐ Eukaryotic cell lines |
| ☒ | ☐ Palaeontology and archaeology |
| ☐ | ☒ Animals and other organisms |
| ☒ | ☐ Clinical data |
| ☒ | ☐ Dual use research of concern |
| ☒ | ☐ Plants |

## Methods

| n/a | Involved in the study |
|---|---|
| ☐ | ☒ ChIP-seq |
| ☐ | ☒ Flow cytometry |
| ☐ | ☐ MRI-based neuroimaging |

## Antibodies

Antibodies used

For immunofluorescence, the following antibodies were used: rabbit anti-CCAAT/enhancer binding protein alpha (C/EBPA, 1:500, 8178P, Cell Signaling Technology), chicken anti-green fluorescent protein (GFP, 1:5000, AB13970, Abcam), rabbit anti-NK homeobox 2-1 (NKX2-1, 1:1000, sc-13040, Santa Cruz), rabbit anti-pro-surfactant protein C (SFTPC, 1:1000, AB3786, Millipore), goat anti-SOX9 (SOX9, 1:1000, AF3075, R&D Systems), rabbit ani-SOX9 (SOX9, 1:1000, AB5535, Millipore). Goat Anti-Mouse Il-33 (IL33, 1:500, R&D, AF3626). rabbit antihomeodomain only protein (HOPX, 1:500, sc-30216, Santa Cruz). mouse anti-homeodomain only protein (HOPX, 1:250, sc-398703 AF647, Santa Cruz). rat anti-KI67 (KI67, 1:1000, 14-5698-82, Invitrogen), guinea pig anti-lysosomal associated membrane protein 3 (LAMP3, 1:500, 391005, SySy), rat anti-epithelial cadherin (ECAD, 1:1000, 13190, Invitrogen).

The following antibodies were used for FACS: PE/Cy7 rat anti-CD45 (CD45, 1:250, 103114, BioLegend), PE rat anti-epithelial cadherin (ECAD, 1:250, 147304, BioLegend), BV421 rat antiepithelial cell adhesion molecule (EPCAM, 1:250, 118225, BioLegend), and AF647 rat antiIntercellular adhesion molecule 2 (ICAM2, 1:250, A15452, Thermo Fisher).

The following antibodies were used for chromatin immunoprecipitation: rabbit anti-NK Homeobox 2-1 (NKX2-1, 1 µg per reaction, ab133737, Abcam) and rabbit anti-CEBPA (C/EBPα, (D56F10) XP, 1 µg per reaction, Cell Signaling Technology).

Validation

Antibodies used in our study were validated as noted by their suppliers and prior publication patterns. CEBPA antibodies were additionally validated through staining mouse lungs and observing expected co-localization with other markers and observing the expected staining pattern (nuclear).

## Animals and other research organisms

Policy information about studies involving animals; ARRIVE guidelines recommended for reporting animal research, and Sex and Gender in Research

| | |
|---|---|
| Laboratory animals | All mouse strains used in this study were of C57BL/6N; C57BL/6J mixed genetic backgrounds. Whenever possible, littermate control and mutant mice were used. All experiments were conducted on mixed populations of male and female. The animals were housed at 22C, 45% humidity, and 12-12 hour light-dark cycle conditions. CebpaF/F were obtained from the Jackson Laboratory (stock #006447 The ages described at manuscript as follows RosaSun1GFP/+; SftpcCreER/+; CebpaF/F were sampled at P7, P8, P9,P10, P11, P17, 5-wk and 8-wk, 14-wk. RosaSun1GFP/+; SftpcCreER/+ were sampled at P2, 6-wk and 7-wk. |
| Wild animals | No wild animals were used in this study. |
| Reporting on sex | All experiments were conducted on mixed populations of males and females. For all scMultiome experiments in the study, we used 1 male and 1 female per sample. |
| Field-collected samples | This study did not involve field-collected samples. |

| | |
|---|---|
| Ethics oversight | All mice were housed in the MD Anderson facility and the proposed studies was performed following all federal regulations on the use of animals in research and have been approved by the Institutional Animal Care and Use Committee at the University of Texas MD Anderson Cancer Center. |

Note that full information on the approval of the study protocol must also be provided in the manuscript.

# Plants

| | |
|---|---|
| Seed stocks | *Report on the source of all seed stocks or other plant material used. If applicable, state the seed stock centre and catalogue number. If plant specimens were collected from the field, describe the collection location, date and sampling procedures.* |
| Novel plant genotypes | *Describe the methods by which all novel plant genotypes were produced. This includes those generated by transgenic approaches, gene editing, chemical/radiation-based mutagenesis and hybridization. For transgenic lines, describe the transformation method, the number of independent lines analyzed and the generation upon which experiments were performed. For gene-edited lines, describe the editor used, the endogenous sequence targeted for editing, the targeting guide RNA sequence (if applicable) and how the editor was applied.* |
| Authentication | *Describe any authentication procedures for each seed stock used or novel genotype generated. Describe any experiments used to assess the effect of a mutation and, where applicable, how potential secondary effects (e.g. second site T-DNA insertions, mosiacism, off-target gene editing) were examined.* |

# ChIP-seq

## Data deposition

- [x] Confirm that both raw and final processed data have been deposited in a public database such as GEO.
- [x] Confirm that you have deposited or provided access to graph files (e.g. BED files) for the called peaks.

| | |
|---|---|
| Data access links<br>*May remain private before publication.* | GSE247271 |

Files in database submission

| | |
|---|---|
| GSM7886399 | P2 AT2 cell CEBPA ChIP-seq rep1 |
| GSM7886400 | P2 AT2 cell CEBPA ChIP-seq rep2 |
| GSM7886401 | P2 AT2 cell CEBPA ChIP-seq rep3 |
| GSM7886402 | 6-wk AT2 cell CEBPA ChIP-seq rep1 |
| GSM7886403 | 6-wk AT2 cell CEBPA ChIP-seq rep2 |
| GSM7886404 | 6-wk AT2 cell CEBPA ChIP-seq rep3 |
| GSM7886405 | P2 AT2 cell NKX2-1 ChIP-seq rep1 |
| GSM7886406 | P2 AT2 cell NKX2-1 ChIP-seq rep2 |
| GSM7886407 | 7-wk control AT2 cell NKX2-1 ChIP-seq rep1 |
| GSM7886408 | 7-wk control AT2 cell NKX2-1 ChIP-seq rep2 |
| GSM7886409 | 7-wk mutant AT2 cell NKX2-1 ChIP-seq rep1 |
| GSM7886410 | 7-wk mutant AT2 cell NKX2-1 ChIP-seq rep2 |
| GSM7886411 | P8 control AT2 cell NKX2-1 ChIP-seq rep1 |
| GSM7886412 | P8 control AT2 cell NKX2-1 ChIP-seq rep2 |
| GSM7886413 | P8 mutant AT2 cell NKX2-1 ChIP-seq rep1 |
| GSM7886414 | P8 mutant AT2 cell NKX2-1 ChIP-seq rep2 |
| GSM7886417 | E14.5 whole lung NKX2-1 ChIP-seq rep1 |
| GSM7886418 | E14.5 whole lung NKX2-1 ChIP-seq rep2 |
| GSM7886421 | 7-wk ctrl AT2 input rep1 |
| GSM7886422 | 7-wk mutAT2 input rep2 |
| GSM7886423 | P8 ctrl AT2 input rep1 |
| GSM7886424 | P8 ctrl AT2 input rep2 |
| GSM7886425 | P8 mutant AT2 input rep1 |
| GSM7886426 | P8 mutant AT2 input rep2 |
| GSM7886427 | P2 ctrl AT2 input rep1 |
| GSM7886428 | P2 ctrl AT2 input rep2 |
| GSM7886433 | 6-wk AT2 Input rep1 |
| GSM7886434 | 6-wk AT2 Input rep2 |
| GSM7886435 | E14.5 whole lung Input ChIP-seq rep1 |
| GSM7886436 | E14.5 whole lung Input ChIP-seq rep2 |

| | |
|---|---|
| Genome browser session<br>(e.g. UCSC) | not needed. |

## Methodology

| | |
|---|---|
| Replicates | At least 2 replicates were used |

| | |
|---|---|
| Sequencing depth | At least 10 million uniquely mapped, high quality, non duplicated, 75bp reads (single-end for ChIP-seq and paired-end for ATAC-seq) were used. |
| Antibodies | The following antibodies were used for chromatin immunoprecipitation: rabbit anti-NK Homeobox 2-1 (NKX2-1, 1 µg per reaction, ab133737, Abcam) and rabbit anti-CEBPA (C/EBPα, (D56F10) XP, 1 µg per reaction, Cell Signaling Technology). |
| Peak calling parameters | Fastqc: fastqc -f fasta -t 8 sample.fastq<br>Trimmomatic: java -jar $TRIMMOMATIC/trimmomatic-0.33.jar SE -phred33 -threads 6 sample.fastq SLIDINGWINDOW:4:15 MINLEN:21<br>Bowtie: bowtie -p 24 -k 1 -v 1 -m 1 -S ~/mm10.bt sample.fastq |
| Data quality | We followed the ENCODE standard and recommended software settings for data processing, and visually verified at least 20 identified peaks in each sample. The numbers of peaks in each sample are listed below<br><br>GEO name sample name FDR 5% total peaks FDR 5% logfc 5 peaks<br>GSM7886399 P2 AT2 cell CEBPA ChIP-seq rep1 11461 11200<br>GSM7886400 P2 AT2 cell CEBPA ChIP-seq rep2 15307 14013<br>GSM7886401 P2 AT2 cell CEBPA ChIP-seq rep3 9438 9101<br>GSM7886402 6-wk AT2 cell CEBPA ChIP-seq rep1 10859 10303<br>GSM7886403 6-wk AT2 cell CEBPA ChIP-seq rep2 9507 9056<br>GSM7886404 6-wk AT2 cell CEBPA ChIP-seq rep3 14530 13119<br>GSM7886405 P2 AT2 cell NKX2-1 ChIP-seq rep1 37549 33264<br>GSM7886406 P2 AT2 cell NKX2-1 ChIP-seq rep2 54618 50439<br>GSM7886407 7-wk control AT2 cell NKX2-1 ChIP-seq rep1 64206 54897<br>GSM7886408 7-wk control AT2 cell NKX2-1 ChIP-seq rep2 68907 55134<br>GSM7886409 7-wk mutant AT2 cell NKX2-1 ChIP-seq rep1 56028 45969<br>GSM7886410 7-wk mutant AT2 cell NKX2-1 ChIP-seq rep2 77853 60800<br>GSM7886411 P8 control AT2 cell NKX2-1 ChIP-seq rep1 65596 52128<br>GSM7886412 P8 control AT2 cell NKX2-1 ChIP-seq rep2 79783 65993<br>GSM7886413 P8 mutant AT2 cell NKX2-1 ChIP-seq rep1 74224 54981<br>GSM7886414 P8 mutant AT2 cell NKX2-1 ChIP-seq rep2 55460 48147<br>GSM7886417 E14.5 whole lung NKX2-1 ChIP-seq rep1 66653 38042<br>GSM7886418 E14.5 whole lung NKX2-1 ChIP-seq rep2 40744 26566 |
| Software | ChIP-seq were analyzed using using Fastqc (0.11.8), Trimmomatic (0.33), Bowtie (2.4.1), Picard (2.9.0), Samtools (1.15), MACS2 ( 2.4.1), Homer (4.10), and Diffbind (3.4.11). Custom script is provided in the Supplementary Software File 1 and individual commands are also references in the methods section of this study. |

# Flow Cytometry

## Plots

Confirm that:

- [x] The axis labels state the marker and fluorochrome used (e.g. CD4-FITC).
- [x] The axis scales are clearly visible. Include numbers along axes only for bottom left plot of group (a 'group' is an analysis of identical markers).
- [x] All plots are contour plots with outliers or pseudocolor plots.
- [x] A numerical value for number of cells or percentage (with statistics) is provided.

## Methodology

| | |
|---|---|
| Sample preparation | see methods |
| Instrument | Both cells and nuclei were sorted by BD FACSAria II cell sorter |
| Software | FlowJo (version 10) |
| Cell population abundance | see methods and Supplementary Figure 5A and Supplementary Figure 6A |
| Gating strategy | see methods and Supplementary Figure 5A and Supplementary Figure 6A |

- [x] Tick this box to confirm that a figure exemplifying the gating strategy is provided in the Supplementary Information.

# Magnetic resonance imaging

## Experimental design

| | |
|---|---|
| Design type | *Indicate task or resting state; event-related or block design.* |
| Design specifications | *Specify the number of blocks, trials or experimental units per session and/or subject, and specify the length of each trial or block (if trials are blocked) and interval between trials.* |
| Behavioral performance measures | *State number and/or type of variables recorded (e.g. correct button press, response time) and what statistics were used to establish that the subjects were performing the task as expected (e.g. mean, range, and/or standard deviation across subjects).* |

## Acquisition

| | |
|---|---|
| Imaging type(s) | *Specify: functional, structural, diffusion, perfusion.* |
| Field strength | *Specify in Tesla* |
| Sequence & imaging parameters | *Specify the pulse sequence type (gradient echo, spin echo, etc.), imaging type (EPI, spiral, etc.), field of view, matrix size, slice thickness, orientation and TE/TR/flip angle.* |
| Area of acquisition | *State whether a whole brain scan was used OR define the area of acquisition, describing how the region was determined.* |

Diffusion MRI ☐ Used ☐ Not used

## Preprocessing

| | |
|---|---|
| Preprocessing software | *Provide detail on software version and revision number and on specific parameters (model/functions, brain extraction, segmentation, smoothing kernel size, etc.).* |
| Normalization | *If data were normalized/standardized, describe the approach(es): specify linear or non-linear and define image types used for transformation OR indicate that data were not normalized and explain rationale for lack of normalization.* |
| Normalization template | *Describe the template used for normalization/transformation, specifying subject space or group standardized space (e.g. original Talairach, MNI305, ICBM152) OR indicate that the data were not normalized.* |
| Noise and artifact removal | *Describe your procedure(s) for artifact and structured noise removal, specifying motion parameters, tissue signals and physiological signals (heart rate, respiration).* |
| Volume censoring | *Define your software and/or method and criteria for volume censoring, and state the extent of such censoring.* |

## Statistical modeling & inference

| | |
|---|---|
| Model type and settings | *Specify type (mass univariate, multivariate, RSA, predictive, etc.) and describe essential details of the model at the first and second levels (e.g. fixed, random or mixed effects; drift or auto-correlation).* |
| Effect(s) tested | *Define precise effect in terms of the task or stimulus conditions instead of psychological concepts and indicate whether ANOVA or factorial designs were used.* |

Specify type of analysis: ☐ Whole brain ☐ ROI-based ☐ Both

| | |
|---|---|
| Statistic type for inference (See Eklund et al. 2016) | *Specify voxel-wise or cluster-wise and report all relevant parameters for cluster-wise methods.* |
| Correction | *Describe the type of correction and how it is obtained for multiple comparisons (e.g. FWE, FDR, permutation or Monte Carlo).* |

## Models & analysis

| n/a | Involved in the study |
|---|---|
| ☐ | ☐ Functional and/or effective connectivity |
| ☐ | ☐ Graph analysis |
| ☐ | ☐ Multivariate modeling or predictive analysis |

| | |
|---|---|
| Functional and/or effective connectivity | *Report the measures of dependence used and the model details (e.g. Pearson correlation, partial correlation, mutual information).* |
| Graph analysis | *Report the dependent variable and connectivity measure, specifying weighted graph or binarized graph, subject- or group-level, and the global and/or node summaries used (e.g. clustering coefficient, efficiency, etc.).* |
| Multivariate modeling and predictive analysis | *Specify independent variables, features extraction and dimension reduction, model, training and evaluation metrics.* |
